# Pilot Study of Dose–Response Effects of Exercise on Change in C-Reactive Protein, Cortisol, and Health-Related Quality of Life Among Cancer Survivors

**DOI:** 10.1089/biores.2018.0003

**Published:** 2018-05-01

**Authors:** Jeanette M. Ricci, Victoria Flores, Isabela Kuroyama, Arash Asher, Heather P. Tarleton

**Affiliations:** ^1^ Department of Health and Human Sciences, Loyola Marymount University, Los Angeles, California.; ^2^ Department of Kinesiology, California State University, Long Beach, California.; ^3^ Department of Psychology, Loyola Marymount University, Los Angeles, California.; ^4^ Cancer Survivorship and Rehabilitation, Cedars-Sinai Samuel Oschin Comprehensive Cancer Institute, Los Angeles, California.

**Keywords:** aerobic and resistance training, cancer survivors, c-reactive protein, cortisol, fatigue, health-related quality of life

## Abstract

Fatigue, stress, and depression contribute to poor health-related quality of life (HRQoL) among cancer survivors. This study examined the effects of combined aerobic and resistance training (CART) on HRQoL and biomarkers of stress. Cancer survivors (*n* = 76, 91% female, 39% breast cancer, 32% gynecologic cancer) were enrolled in CART for three 60-min sessions, weekly, for 26 weeks. Participants completed the National Institutes of Health's Patient Reported Outcomes Measurement Information System (NIH PROMIS) fatigue assessment and the SF-36. Cortisol and c-reactive protein (CRP) were assessed using volunteered blood specimens. Baseline fatigue scores were worse for participants completing treatment within the last year, compared to long-term survivors [*F* = (2, 59) = 3.470, *p* = 0.038]. After 26 weeks, fatigue scores improved by a noteworthy two points [*M* = 52.72, standard deviation, SD = 10.10 vs. *M* = 50.67, SD = 10.14; *t*(48) = 1.7145, *p* = 0.092]. Pre- to postintervention improvements in bodily pain [*M* = 50.54, SD = 9.51 vs. *M* = 48.20, SD = 10.07; *t*(33) = 2.913, *p* = 0.006] and limitations in social functioning [*M* = 50.60, SD = 9.17 vs. *M* = 47.75, SD = 11.66; *t*(33) = 2.206, *p* = 0.034], as well as a mean decrease of 1.64 ± 10.11 mg/L in CRP levels [*t*(107) = 1.261, *p* = 5.965], were observed. Participants within 1 year of treatment completion experienced greater improvements in post CRP levels compared to those who had treatment 1–4 years (*p* = 0.030) and 5 or more years ago (*p* = 0.023). Physical functioning, fatigue, fear/anxiety, social role satisfaction, and CRP levels improved following participation in this exercise intervention. Oncologists should consider recommending CART as soon as medically feasible following the cessation of cancer treatment.

## Background

Medical advances will lead to a larger population of aging adults who can be classified as cancer survivors and who may face challenges such as worsening health-related quality of life (HRQoL).^1^ After treatment, survivors experience depression, fatigue and poor motivation to engage physically in daily activities and may develop anxiety and fear with as many as 19% of survivors reportedly meeting the criteria for post-traumatic stress disorder.^2^ Survivors with untreated fatigue and stress are less likely to adhere to recommended cancer surveillance and are less likely to engage in health-promoting activities.^3^

Physical activity is linked to increased functional capacity, improved mood, increased immune function, decreased fatigue, improved health perceptions, and improved HRQoL.^4,5^ From a psychosocial perspective, participating in physical activity provides a positive feedback loop as the cancer survivor sees his/her body respond and engage successfully in exercise.^6^ From a biological perspective, exercise has been suggested to reduce stress and chronic inflammation.^7^ Stress and chronic inflammation are indicated by high levels of cortisol and c-reactive protein (CRP), respectively. Abnormally high cortisol levels can also disrupt the hypothalamic-pituitary-adrenal axis and contribute to an increase in CRP.^8^ High levels of CRP are associated with poor HRQoL, impaired physical function, and fatigue in cancer survivors.^9,10^

### The Improving Physical Activity After Cancer Treatment Study

Previously published studies of exercise interventions in cancer survivors focused on one exercise modality, often in a short (e.g., 12-week) intervention period.^4^ This study's exercise prescription uses a combined aerobic and resistance training (CART) protocol with the addition of core strengthening and flexibility over a 26-week intervention period.

Data collected and pooled from two cohorts of cancer survivors that participated in the 26-week exercise program were used to determine whether there is a dose–response relationship between participation in exercise training and changes in self-reported HRQoL. The study also aimed to strengthen the existing body of literature by adding pre- and postintervention measurement of cortisol and CRP to assess whether there is a dose–response relationship between participation in exercise training and change in physiological stress and inflammation, respectively.

## Materials and Methods

### Study population

The Improving Physical Activity After Cancer Treatment (IMPAACT) Study was designed as a prospective study of the effect of participation in a 26-week exercise training program on the health of cancer survivors. The study was approved by the Loyola Marymount University Institutional Review Board (LMU IRB 2014 SP 27 and LMU IRB 2015 SP 23) and the California Health and Human Services Agency Institutional Review Board (Protocol ID 14-02-1507) in accordance with the Helsinki Declaration of 1975, as revised in 1983, and the Declaration of the World Medical Association.

Two cohorts of cancer survivors were recruited (Cohort #1, *n* = 33 and Cohort #2, *n* = 43) at two timepoints by convenience sampling using survey distribution in Los Angeles County, cancer survivor support group referral, and physician referral. Written informed consent was ascertained before study enrollment. Individuals had to meet the following criteria to be eligible for participation: primary diagnosis of cancer, treatment completed at any point before the study, and physical ability to partake in moderate-intensity weight-bearing exercise.

Participants were excluded from the study if they presented with any of the following conditions: pregnancy, stroke, or heart attack within past 6 months and lymphedema. Participants were asked to inform their physicians of their planned participation and to review the exercise and assessment protocols with their physicians before the intervention.

### Assessments

#### Demographics, NIH PROMIS, and SF-36

At recruitment, participants self-reported demographics, comorbidities, health behaviors, and perception of physical activity level. Participants' height and weight were collected by trained researchers to compute body mass index (BMI), in addition to completing waist circumference measurements. Participants' HRQoL was assessed using the fatigue domain of the National Institutes of Health's Patient Reported Outcomes Measurement Information System, version 1.0 (NIH PROMIS) and the 36-item short-form survey, version 2.0 (SF-36).

Participants completed the NIH PROMIS fatigue assessment, a subcomponent of the physical function domain with applicability in diverse samples of cancer patients.^11^ The NIH PROMIS fatigue assessment has been validated against the Fatigue Symptom Inventory and the Functional Assessment of Chronic Illness Therapy-Fatigue to measure fatigue severity and disruptiveness in cancer patients and cancer survivors.^12,13^

A three-to-five point change in fatigue *T-score* has been estimated as clinically meaningful for cancer patients and across cancer type.^12,14^ Administration of the NIH PROMIS fatigue survey included computerized adaptive testing (CAT) for Cohort #1 and the eight item short-form paper (version 1.0, Fatigue 8a) for Cohort #2. The short form has been shown to be a reliable and precise alternative for CAT regarding psychometric analysis of fatigue, with no differential effect on the validity of scores across mode of assessment.^15^

The psychometric properties of the SF measures on various cancer patients and survivors have been assessed and shown to have good internal consistency and validity.^16^ Clinically meaningful and statistically significant postintervention differences following an exercise program have been reported as a change by 4–15 *T-score* points.^17,18^

#### Biomarkers

Blood specimens were collected by a licensed phlebotomist between 7 and 9am following an overnight fast at baseline, midpoint, and postintervention to measure serum concentrations of cortisol and CRP. Serum was isolated by trained laboratory technicians at the LMU Biomedical Sciences Laboratory and stored at −80°C. Cortisol (Abcam 108665; intra-assay ≤9.0% coefficient of variation [CV], interassay ≤9.0% CV) and CRP (Millipore HNDG2MAG-36; intra-assay <10% CV, interassay <15% CV) were analyzed from isolated serum at the Norris Comprehensive Cancer Center at the University of Southern California. All blood specimens were deidentified and coded using a nonpersonal, randomly assigned study identification number.

### Intervention

All assessments and exercise sessions were supervised by a registered clinical exercise physiologist and by an American College of Sports Medicine (ACSM) certified health fitness specialist. Participants completed 60-min sessions, three times a week for 26 weeks, that included 15 min of aerobic walking/running at 35–85% heart rate reserve, 30 min of whole body circuit training, and 15 min of flexibility and core training as prescribed in accordance with the Guidelines for Exercise for Cancer Survivors from the ACSM.^19^ Each participant wore a Polar heart rate monitor (Polar Electro, Lake Success, NY) for assessments and exercise sessions, and all assessment and training staff were trained in First Aid and certified in cardio-pulmonary resuscitation.

### Data analysis

Data from both cohorts were pooled given the high degree of similarity in the source population for participant recruitment and in data collection and intervention protocols. Analysis of Cohort #1 is based on 33 enrolled participants with 21 (64%) completing the intervention. Analysis of Cohort #2 is based on 43 enrolled participants with 35 (81%) completing the intervention.

Cohort #1 and Cohort #2 were implemented in sequential order and separated by a 3-month gap and 13 participants from Cohort #1 also enrolled in Cohort #2. In pooled analyses, data from these 13 participants in Cohort #2 (30%) were excluded to ensure that each data point is independent and to avoid potential bias from training effects. This yields 63 unique enrolled participants in the pooled analysis with 46 (73%) completing the intervention. Reasons reported for attrition included transportation limitations, work schedules, return to cancer treatment, and post-treatment reconstructive surgery.

Participation in exercise training was recorded in-person at each exercise session and used to calculate a cumulative percent participation in training. For stratified analysis, participation was defined as ≤50% of sessions attended, 51–74% attended, and ≥75% attended. Participants self-reported clinical diagnosis of chronic conditions, which was combined to define comorbidity as presence of 0, 1, 2, or >2 chronic conditions. BMI was divided into normal weight (18.35–24.9; one participant did not reach standard 18.5 cutpoint), overweight (25–29.9), and obese (≥30) categories based on the widely accepted ACSM cutpoints.^20^

Recovery from cancer treatment is nonlinear, and the survivorship experience is unique to each individual.^21^ The survivorship experience has been best articulated as a series of seasons in which a cancer patient moves from an acute stage of diagnosis and treatment initiation, through an extended stage of treatment cessation and navigating treatment-related side effects, and into a permanent stage of lower risk of recurrence but possibly with continued need to manage treatment-related side effects.^22^

The transition from the acute stage to the extended stage is arguably the most difficult as the specialized medical and psychosocial care provided during treatment is withdrawn at treatment cessation.^23^ Survival statistics are typically based on the number of cancer patients that survive from diagnosis to 5- and 10-year marks. These cutpoints, however, do not reflect trajectory of improvement of HRQoL, which is the hallmark of the extended stage of the survivorship experience.^24^

Furthermore, physiological stress and inflammation (CRP, cortisol) can be greatly affected within months of treatment cessation.^25^ Therefore, for this study, time since treatment (TST) was categorized into three groups (<1, 1–4, 5+ years) to more finely stratify the extended stage based on reports of persistence of treatment-related effects from 6 to 12 months after treatment cessation and given the conventional use of 5 years as the transition from the extended to the permanent stage.

All statistical analyses were conducted using SPSS version 23 (IBM Corp., Armonk, NY). Statistical outliers were first determined by SPSS and removed from the data analysis. Results with and without outliers were manually compared using box and normality plots and to identify major deviations in findings and to ensure that no more than 10% of data were removed from analysis by SPSS. Sample sizes were fixed on previous participant pools for cohort 1, 2, and pooled sections. For all statistical and stratified analyses, alpha levels were set to 0.05, and power levels were set to 0.80 (Table 1). Either Cohen's *d* or Pearson's *r* was determined for effect size where analysis of variances (ANOVAs), *t*-tests, and multinomial regressions were significant.

**Table 1.** **Power Analysis (α = 0.05)**

Variables	Statistical test	Effect size	Power
Baseline fatigue, TST	ANOVA	0.323	0.602
Physical activity, baseline fatigue	ANOVA	0.355	0.691
TT, baseline fatigue	ANOVA	0.248	0.491
Baseline WC, baseline fatigue	Unpaired *t*-test	*r* = 0.832 Cohen's *d* = 3.00	0.99
Baseline cortisol, baseline fatigue	Unpaired *t*-test	*r* = 0.382 Cohen's *d* = 0.828	0.847
Baseline CRP, baseline fatigue	Unpaired *t*-test	*r* = 0.780 Cohen's *d* = 2.49	0.99
Baseline fatigue, postintervention fatigue	Unpaired *t*-test	*r* = 0.844 Cohen's *d* = 3.15	0.996
Participation, postintervention fatigue	ANOVA	0.217	0.304
TST, baseline general health	ANOVA	0.468	0.591
TST, baseline bodily pain	ANOVA	0.393	0.443
Baseline CRP, TST	ANOVA	0.209	1.00
BMI, baseline CRP	ANOVA	0.541	1.00
Baseline WC, baseline CRP	Unpaired *t*-test	*r* = 0.780 Cohen's *d* = 2.49	0.98
Postintervention CRP, TST	ANOVA	0.084	0.415
Baseline cortisol, baseline WC	Unpaired *t*-test	*r* = 0.382 Cohen's *d* = 0.828	0.553
Baseline cortisol, comorbidity	ANOVA	0.114	0.079

ANOVA, analysis of variance; BMI, body mass index; CRP, c-reactive protein; TST, time since treatment; TT, treatment type; WC, waist circumference.

Unpaired *t*-tests were used to determine differences between pooled, preintervention PROMIS fatigue scores and waistline circumference, baseline cortisol, baseline CRP, and postintervention fatigue scores. Unpaired *t*-tests were also used in pooled comparisons between waist circumference and baseline CRP and cortisol values. A paired *t*-test was used to compare baseline waist circumference with postintervention waist circumference scores.

One-way ANOVA was used to determine differences between pooled, preintervention PROMIS fatigue scores and TST, perceived physical activity, number of comorbidities, participation, and treatment type categories. Where ANOVAs were significant, Tukey's honest significant difference was used for *post hoc* comparisons. Where homogeneity was violated, Welch's *p* was reported and Games–Howell *post hoc* comparisons were used.

One-way ANOVAs were used for comparisons between TST groups, comorbidity and physical component summary (PCS), mental component summary (MCS), and all eight SF-36 domains. One-way ANOVAs were also used in pooled comparisons between baseline CRP levels with TST, BMI, and waist circumference groups and postintervention CRP levels with TST groups. Finally, one-way ANOVAs were used for baseline cortisol comparisons with TST, BMI, and comorbidity.

For stratified analyses, one-way analysis of covariance was used to determine the effect of covariates (baseline scores) on postintervention measures for fatigue, cortisol, CRP, PCS, MCS, and all eight SF-36 domains while controlling for BMI, comorbidity, TST, cancer treatment type, and race.

## Results

Participants were, on average, college-educated women in their early-to-mid 60s with at least two diagnosed chronic conditions in addition to a cancer diagnosis (Table 2). The majority of participants identified as white, and approximately one-third identified with a historically underrepresented racial minority group. Given that the majority of participants were women, the most represented cancer types were breast and gynecologic cancers (Table 2). Before the intervention, 25.4% participants self-reported their physical activity level as *very good* or *good*, whereas 74.6% of participants reported *okay* or *needs improvement*.

**Table 2.** **Demographics of Participants at Baseline**

Variable	Cohort #1 (*n* = 33)	Cohort #2 (*n* = 43)	*p*
Age (mean years ± SD)	61 ± 12.58	65 ± 7.39	0.080
Sex, male	6 (18%)	1 (2%)	0.017^*^
Sex, female	27 (82%)	42 (98%)	
Race			
White	20 (61%)	29 (67%)	0.585
African American, Hispanic	11 (33%)	10 (23%)	
Other/multiracial	2 (6%)	4 (10%)	
Education			
High school education	2 (6%)	3 (7%)	0.958
Some college	10 (30%)	14 (33%)	
4-Year college education	21 (64%)	26 (60%)	
Employment status			
Employed	9 (27%)	10 (23%)	0.552
Retired	11 (33%)	21 (49%)	
Unemployed	3 (9%)	2 (5%)	
Disabled/other support	10 (31%)	10 (23%)	
Time since treatment (mean years ± SD)	4.30 ± 8.26	6.93 ± 3.84	0.071
Cancer type			
Breast	17 (52%)	13 (30%)	<0.001^*^
Colorectal	7 (21%)	2 (5%)	
Myeloma/lymphoma	3 (9%)	3 (7%)	
Gynecologic	2 (6%)	22 (52%)	
Thyroid	2 (6%)	1 (2%)	
Prostate	1 (3%)	1 (2%)	
Skin	1 (3%)	0 (0%)	
Lung	0 (0%)	1 (2%)	
Diagnosed chronic conditions (mean ± SD)	2.34 ± 0.74	2.37 ± 1.64	0.920

^*^ Statistically significant at α = 0.05.

SD, standard deviation.

### NIH PROMIS fatigue

At baseline, PROMIS fatigue scores were significantly worse for participants completing treatment within the last year compared to those with a TST greater than 5 years (*p* = 0.038; *r* = 0.323), suggesting a greater burden of fatigue among survivors that have more recently completed cancer treatment. Those who perceived their physical activity level as *very good* had significantly better fatigue scores (*p* = 0.039; *r* = 0.355) compared to those who reported *needs improvement*.

Comorbid burden was not associated with fatigue scores, but there was a difference in fatigue scores between no chemotherapy and chemotherapy treatment groups (*p* = 0.043; *r* = 0.248). At baseline, a positive relationship was observed with waist circumference and fatigue (*p* < 0.0001; *r* = 0.832; *d* = 3.00). Fatigue scores were also associated with baseline cortisol (*p* < 0.0001; *r* = 0.382; *d* = 0.828) and CRP levels (*p* < 0.0001; *r* = 0.780; *d* = 2.49).

**FIG. 1.**
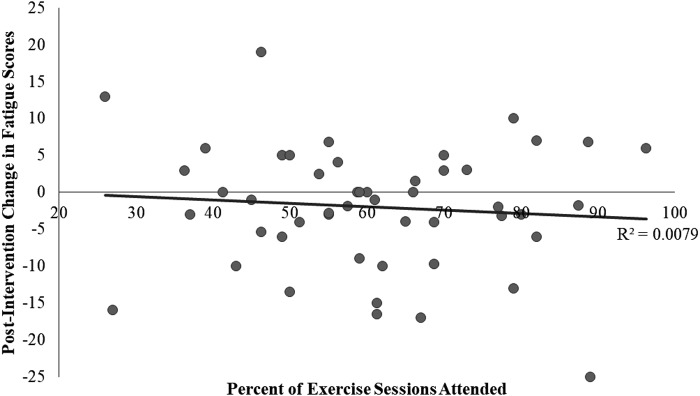
Effect of participation frequency on NIH PROMIS fatigue domain scores after 26 weeks of group exercise, The IMPAACT Study 2014–2016, *n* = 49. IMPAACT, Improving Physical Activity After Cancer Treatment; NIH PROMIS, National Institutes of Health's Patient Reported Outcomes Measurement Information System.

After 26 weeks of exercise intervention, self-reported NIH PROMIS fatigue scores for the pooled cohort improved by a noteworthy two points (*p* = 0.092). There appears to be a dose–response relationship between percent of sessions attended during the intervention and improvement in self-reported fatigue scores (Fig. 1), although results lost significance at an alpha of 0.05 when controlling for baseline scores (*p* = 0.178). In stratified analysis, baseline BMI, comorbidity, TST, cancer treatment type, and race had no observable modifying effect on the relationship between participation and fatigue.

### SF-36 HRQoL survey

Among Cohort #2 participants, baseline TST was inversely related to baseline physical function (*p* = 0.068) and baseline general health (*p* = 0.007; *r* = 0.468), suggesting that those with longer TST had worsened physical function and general health. TST was positively associated with bodily pain (*p* = 0.026; *r* = 0.393), suggesting greater self-reported pain in those furthest from treatment cessation. No observable TST relationship was detected for physical role, vitality, social function, emotional role, mental health, PCS, and MCS. Comorbid burden was inversely related to vitality (*p* = 0.019) and physical function (*p* = 0.0827). However, no associations were identified for comorbid burden and physical role, bodily pain, general health, social function, emotional role, mental health, or MCS.

Participants in Cohort #2 that reported a higher comorbid burden at baseline appeared to experience the greatest improvements in the SF-36 vitality domain after 26 weeks of CART (*p* = 0.019). Significant pre- to postintervention improvements in self-reported bodily pain (*p* = 0.006) and limitations in social functioning (*p* = 0.034) were also observed (Table 3). No significant postintervention changes were identified for the remaining five SF-36 domains (Table 3).

**Table 3.** **SF-36 Subscale and Summary Score Assessments: Preintervention to Postintervention (*n* = 35)**

Subscale measure	Cohort #2 preintervention mean (SD)	Cohort #2 postintervention mean (SD)	Pre–post mean change mean (SD)	*p*
Vitality	51.15 (10.81)	51.40 (10.40)	0.25 (−0.41)	0.890
Bodily pain	50.54 (9.51)	48.20 (10.07)	−2.34 (0.56)	0.006^*^
Mental health	52.96 (7.66)	52.40 (9.91)	−0.56 (2.25)	0.546
Physical functioning	48.73 (7.64)	47.03 (8.75)	−1.70 (1.11)	0.077
Social functioning	50.60 (9.17)	47.75 (11.66)	−2.85 (2.49)	0.034^*^
Emotional roles	50.59 (6.81)	49.50 (10.05)	−1.09 (3.24)	0.441
Physical roles	47.46 (10.84)	46.69 (10.51)	−0.77 (−0.33)	0.659
General health	52.88 (9.91)	54.29 (9.98)	1.41 (−0.07)	0.156
PCS	48.73 (10.14)	48.04 (9.18)	−0.69 (−0.96)	0.457
MCS	52.63 (8.07)	51.82 (10.25)	−0.81 (2.18)	0.466

^*^ Statistically significant at α = 0.05.

MCS, mental component summary; PCS, physical component summary.

**FIG. 2.**
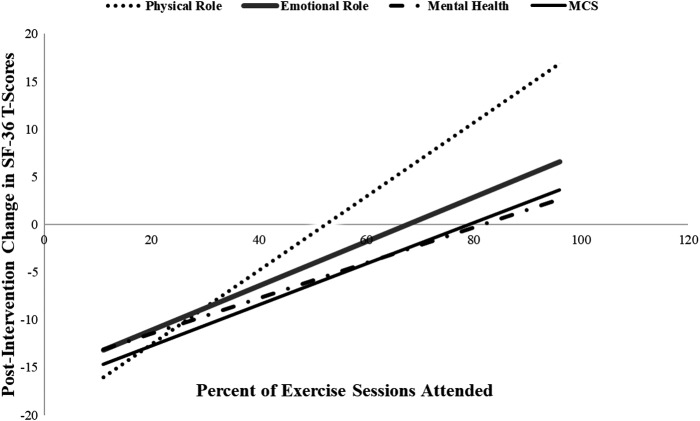
Effect of participation frequency on SF-36 domains after 26 weeks of group exercise, The IMPAACT Study 2015–2016 (Cohort #2), *n* = 35.

Postintervention changes in bodily pain appeared to be mediated by TST (*p* = 0.013), but not modified by baseline BMI, comorbid burden, cancer treatment type, and race. The observed postintervention change in social functioning was not modified by TST. Improvements in all SF-36 domains appeared to be related to frequency of participation in the intervention in a dose–response manner; however, none of the dose–response relationships were significant at an alpha of 0.05 (Fig. 2). Postintervention SF-36 domain scores from Cohort #1 were not different than those from Cohort #2 (Table 4). The mental health and physical health summary scores (MCS and PCS, respectively) did not differ significantly between cohorts after the 26-week intervention (Table 5).

**Table 4.** **SF-36 Subscale and Summary Score Assessments: Cohort Comparison**

Subscale measure	Cohort #1 (*n* = 15) postintervention mean (SD)	Cohort #2 (*n* = 35) postintervention mean (SD)	Post mean differences between cohorts	*p*
Vitality	50.22 (11.0)	51.40 (10.40)	1.18 (−0.6)	0.719
Bodily pain	48.88 (8.51)	48.20 (10.07)	−0.68 (1.56)	0.820
Mental health	51.04 (8.53)	52.40 (9.91)	1.36 (1.38)	0.645
Physical functioning	48.13 (9.38)	47.03 (8.75)	−1.10 (−0.63)	0.691
Social functioning	47.65 (9.17)	47.75 (11.66)	0.10 (2.49)	0.976
Emotional roles	49.44 (7.49)	49.50 (10.05)	0.06 (2.56)	0.983
Physical roles	48.47 (9.05)	46.69 (10.51)	−1.78 (1.46)	0.570
General health	53.91 (9.33)	54.29 (9.98)	0.38 (0.65)	0.900

**Table 5.** **SF-36 Subscale and Summary Score Assessments: Summary Score Comparison**

Postintervention	PCS score mean (SD)	MCS score mean (SD)	*p*
Cohort #1 (*n* = 15)	49.19 (9.32)	50.35 (9.58)	0.739
Cohort #2 (*n* = 35)	48.04 (9.18)	51.82 (10.25)	0.114

### C-reactive protein

At baseline, mean CRP levels were lower in the <1 year TST group compared to the 1–4 year (*Welch's p* = 0.0250) and 5+ year (*Welch's p* = 0.009) TST groups (Table 6). A positive relationship was observed between waist circumference and CRP (*p* < 0.0001; *r* = 0.780; *d* = 2.49) and between BMI and CRP (*Welch's p* < 0.0001; *r* = 0.541) at baseline. However, the relationship between baseline waist circumference and CRP lost significance after controlling for TST (*p* = 0.815).

**Table 6.** **Mean Values of Pre- and Postoutcome Variables and Mean Values of Pre- and Postoutcome Variables by Time Since Treatment Group**

		Pre (0 weeks)		Post (26 weeks)	
Outcome variable	TST group	Mean (SD)	*p*	Mean (SD)	*p*
CRP (mg/L)	—	21.45 (22.82)	—	28.97 (39.13)	0.210
	<1 year	7.27 (4.50)	0.025^a*^	4.73 (3.84)	0.030^a*^
	1–4 years	25.27 (26.76)	0.955^b^	34.91 (40.08)	0.726^b^
	5+ years	23.02 (24.04)	0.009^c*^	25.32 (33.70)	0.023^c*^
Cortisol (ng/mL)	—	162.17 (89.44)	—	173.00 (101.60)	0.556
	<1 year	193.15 (91.62)	0.317^a^	150.10 (42.42)	0.978^a^
	1–4 years	143.01 (66.72)	0.663^b^	62.11 (16.03)	0.987^b^
	5+ years	166.56 (106.20)	0.699^c^	163.33 (76.45)	0.950^c^
Waist circumference (cm)	—	94.94 (17.61)	—	91.54 (15.26)	0.0002^**^
	<1 year	99.30 (14.19)	0.931^a^	90.70 (15.44)	0.991^a^
	1–4 years	97.01 (20.03)	0.896^b^	91.73 (16.67)	1.000^b^
	5+ years	94.47 (17.98)	0.715^c^	90.68 (15.33)	0.977^c^

^a^ *Post hoc* comparison between <1 and 1–4 year groups.

^b^ *Post hoc* comparison between 1–4 and 5+ year groups.

^c^ *Post hoc* comparison difference between <1 and 5+ year groups.

^*^ Significant difference between *post hoc* comparison (*p* < 0.05).

^**^ Significant difference between pre- and postoutcome variable (*p* < 0.05).

Collectively, this cross-sectional analysis suggests a synergism among the CRP, TST, and waist circumference variables among cancer survivors that differ from the expected linear CRP and waist circumference (central adiposity) relationship observed in cancer-free adults. There was no observable relationship between age and CRP levels or between comorbid burden and CRP levels at baseline.

**FIG. 3.**
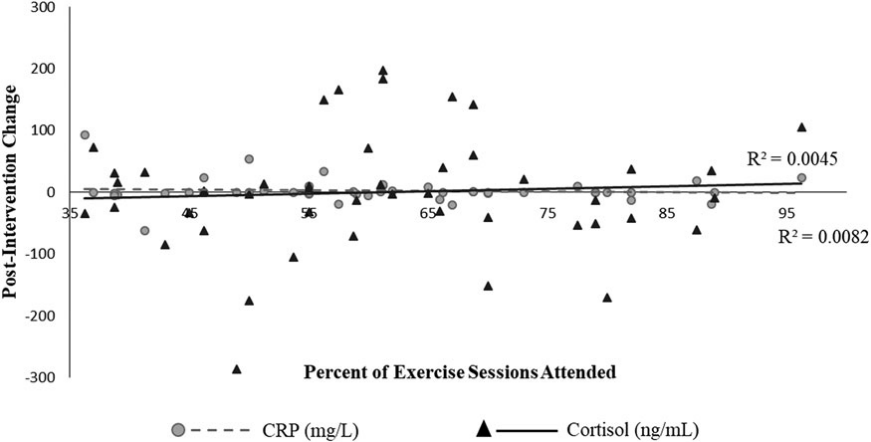
Effect of CART participation frequency on CRP and cortisol biomarkers after 26 weeks of group exercise, The IMPAACT Study 2014–2016, *n* = 46. CART, combined aerobic and resistance training; CRP, c-reactive protein.

Following the 26-week intervention, waist circumference decreased on average by 3.40 cm (*p* = 0.0002). A mean decrease of 1.64 ± 10.11 mg/L in CRP levels was also observed in the combined cohort (Fig. 3). Participants who were within 1 year of treatment completion experienced greater improvements in post CRP levels compared to those who had treatment from 1 to 4 years (*Welch's p* = 0.030) and 5 or more years ago (*Welch's p* = 0.023). The dose–response relationship between participation and decreases in CRP was not mediated by treatment type.

### Cortisol

In the pooled analysis, baseline cortisol levels were not associated with TST nor found to be significantly different than postintervention cortisol levels (Table 6). No association was detected between baseline cortisol and BMI category, but a positive correlation was observed with waist circumference (*p* < 0.0001; *r* = 0.382; *d* = 0.828), suggesting a role for central adiposity. A positive association was observed between comorbid burden and baseline cortisol levels (*p* < 0.0001; *r* = 0.114), indicating that participants with multiple comorbidities were more likely to experience high cortisol levels.

## Discussion

### Summary of findings

At baseline, 75% of this study's participants reported an average or below average perception of their physical activity levels. In addition, participants with poorer perceptions of their physical activity levels were more likely to have completed treatment recently. This finding is consistent with Gjerset et al. who found that among cancer survivors experiencing chronic fatigue, 86% desired physical training compared to 65% of survivors without chronic fatigue.^26^

Self-reported fatigue was particularly noteworthy among participants who received chemotherapy and positively associated with increased baseline body weight and cortisol levels. It was also noted that participants with higher self-reported comorbidity had higher cortisol levels and decreased vitality and physical function, observations consistent with poorer quality of life due to multiple medical conditions and the stress of navigating poorer health. However, participants with multiple chronic conditions experienced the most noteworthy postintervention improvement in bodily pain, social functioning, and vitality.

Self-reported NIH PROMIS fatigue scores improved marginally with increased participation in the 26-week intervention by a noteworthy two points. Participants also improved self-reported bodily pain and limitations in social functioning following 26 weeks of CART. These findings support previous studies in the adult breast cancer survivor population that found improvements in SF-36 domains following a resistance training program,^27^ an aerobic exercise program,^18^ or an exercise education program.^17^

### The role of social support

The peer affirmation and social support received during this group exercise intervention may have positively influenced the perception of their health and physical ability. Social support could provide a beneficial effect due to continued positive experiences and socially rewarded actions in the exercise intervention. Support from a social network is related to positive physical health outcomes through neuroendocrine changes.^28^

This social support may have also contributed to the high program adherence demonstrated throughout the intervention. Fraser and Spink showed high exercise intervention compliance in adult females that valued the role of social support.^29^ Likewise, Nock et al. interviewed breast cancer survivors following a group exercise program and found that social support was one of three primary motivators to engage in the program.^30^

### Mediators of stress-related biomarkers

In addition to examining the potential for improving HRQoL and perceived barriers with exercise among cancer survivors, this study also contributes new insight into how the anti-inflammatory effects of exercise and reduction of CRP may be mediated by body composition and TST.^5^ The intervention appeared to have the greatest impact on reducing CRP levels among participants that had most recently completed treatment, which underscores the greater opportunity for improvement with earlier intervention post-treatment. CRP levels also responded positively to exercise intervention among those participants with normal baseline levels of CRP, which is consistent with previously published studies.^31,32^

Exercise did not appear to have a substantial effect on CRP among those cancer survivors with above normal baseline levels of CRP. The abnormal CRP levels (>3.0 mg/L) are indicative of chronic inflammation and might be related to a participant's treatment regimen, body weight, or a combination of both.^33^ This suggests a need to further examine CRP levels specifically in cancer survivors to establish a separate scale for average CRP level by body weight, TST, treatment type, and cancer type.

Currently, levels above 10 mg/mL are considered indicative of acute inflammation, and levels above 25 mg/mL are excluded from analysis as too extreme.^34^ However, this exclusionary approach may not be ideal for a population of cancer survivors and may limit the full elucidation of the CRP response pathway. In addition, before the intervention cortisol levels were positively associated with waist circumference. Thus, more attention to the influence of body composition on stress-related inflammation is needed given that the body composition of most adults, especially postmenopausal women, shifts toward increases in visceral fat mass with aging.

Given that previously published studies of different exercise modalities report a mixture of outcomes regarding CRP and inflammatory mediators,^35^ it is possible that survivors with unique biomarker profiles may need more specific exercise prescriptions for aerobic and resistance training.

## Conclusions

This study had multiple strengths, including above average study compliance rates, completion of both perceived and physiological measures of fatigue and stress, and inclusion of covariates, including comorbid burden and TST in analysis. The length of this intervention was longer than the majority of previous cancer survivorship studies and included a novel group exercise design.

Acceptability to the intervention is demonstrated by an average participation of 73% for the pooled cohort, despite complications that would be expected for an older and medically complex population. The difference in participation between the two cohorts might be explained by times that sessions were offered. Cohort #1 participants were given the option of participating in either a noon or 5pm session. Cohort #2 participants were given the option of participating in either a 10am or a noon session, and since Cohort #2 had more retired individuals, this may have helped with attrition.

Despite the encouraging results demonstrated in the study, there are limitations that must be considered. First, results may not be generalizable to other cancer populations given the small sample size and narrow range of cancer types included in this study. A control group may have strengthened our findings, however, the primary aim was to determine if there was a dose-dependent response between participation and change in outcome measures. This allowed comparison between frequently attending and low attending participants as an internal control/stratum.

In conclusion, this study suggests that starting an exercise program would be of greatest benefit to those patients who have most recently completed treatment and who have received chemotherapy in particular. The findings from this study suggest a complex relationship between treatment, body weight, fatigue, and biomarkers of stress and inflammation. The intricate mapping of the relationships between stress-related inflammation, exercise interventions, and perceived HRQoL among cancer survivors is still being developed, and larger studies will be needed to establish the temporality and directionality of these relationships.

## Abbreviations Used

ACSM: American College of Sports Medicine
ANOVA: analysis of variance
BMI: body mass index
CART: combined aerobic and resistance training
CAT: computerized adaptive testing
CRP: c-reactive protein
HRQoL: health-related quality of life
IMPAACT: Improving Physical Activity After Cancer Treatment
MCS: mental component summary
NIH PROMIS: National Institutes of Health's Patient Reported Outcomes Measurement Information System
PCS: physical component summary
SD: standard deviation
TST: time since treatment
